# Short Chain Fatty Acids Modulate the Growth and Virulence of Pathosymbiont *Escherichia coli* and Host Response

**DOI:** 10.3390/antibiotics9080462

**Published:** 2020-07-30

**Authors:** Shiying Zhang, Belgin Dogan, Cindy Guo, Deepali Herlekar, Katrina Stewart, Ellen J. Scherl, Kenneth W. Simpson

**Affiliations:** 1 Department of Clinical Sciences, College of Veterinary Medicine, Cornell University, Ithaca, NY 14853, USA; sz11@cornell.edu (S.Z.); belgin.dogan@cornell.edu (B.D.); cg567@cornell.edu (C.G.); deepali.eevmc@gmail.com (D.H.); katrina_harley@yahoo.com.au (K.S.); 2 Plainfield Animal Hospital, 2215 Park Ave., South Plainfield, NJ 07080, USA; 3 Sage Veterinary Centers, 1410 Monument Blvd, Concord, CA 94520, USA; 4 The Roberts IBD Center, Weill-Cornell Medical College, 71st and York, New York, NY 10065, USA; ejs2005@med.cornell.edu

**Keywords:** short chain fatty acids (SCFA), inflammatory bowel disease (IBD), Crohn’s disease (CD), adherent invasive *E. coli* (AIEC), colorectal cancer (CRC), pathosymbiont, gene expression, motility, invasion, host

## Abstract

Short chain fatty acids (SCFA), principally acetate, propionate, and butyrate, are produced by fermentation of dietary fibers by the gut microbiota. SCFA regulate the growth and virulence of enteric pathogens, such as enterohemorrhagic *E. coli* (EHEC), *Klebsiella* and *Salmonella*. We sought to investigate the impact of SCFA on growth and virulence of pathosymbiont *E. coli* associated with inflammatory bowel disease (IBD) and colorectal cancer (CRC), and their role in regulating host responses to bacterial infection in vitro. We found that under ileal conditions (pH = 7.4; 12 mM total SCFA), SCFA significantly (*p* < 0.05) potentiate the growth and motility of pathosymbiont *E. coli*. However, under colonic conditions (pH = 6.5; 65 to 123 mM total SCFA), SCFA significantly (*p* < 0.05) inhibit growth in a pH dependent fashion (up to 60%), and down-regulate virulence gene expression (e.g., *fliC, fimH, htrA, chuA, pks)*. Functional analysis reveals that colonic SCFA significantly (*p* < 0.05) inhibit *E. coli* motility (up to 95%), infectivity (up to 60%), and type 1 fimbria-mediated agglutination (up to 50%). In addition, SCFA significantly (*p* < 0.05) inhibit the activation of NF-*κ*B, and IL-8 production by epithelial cells. Our findings provide novel insights on the role of the regional chemical microenvironment in regulating the growth and virulence of pathosymbiont *E. coli* and opportunities for therapeutic intervention.

## 1. Introduction

Short-chain fatty acids (SCFA), primarily acetate, propionate, and butyrate, are produced by microbial fermentation of undigested carbohydrates and dietary fibers [1,2]. The amount and type of SCFA in the intestine are influenced by dietary intake, particularly non-digestible carbohydrates, protein and fat [3,4], and the composition of the gut microbiota [3,5]. The main producers of SCFA are Firmicutes and Bacteroidetes, the two most abundant phyla in human intestine [2,4]. Bacteroidetes produce mainly acetate and propionate, while Firmicutes produce butyrate [6,7]. Acetate is the most abundant SCFA in the gut and is produced from acetyl-CoA through glycolysis; butyrate and propionate are produced from both carbohydrate metabolism (glycolysis) and metabolisms of fatty and amino acids [8]. The concentration gradient of SCFA in the intestine increases from the small to large intestine [9,10], paralleling the density of resident microbiota. Depending on diet, the concentration of total SCFA in the large bowel ranges from 60 to 150 mM, with a molar ratio of 60:20:20 for acetate, propionate, and butyrate [9,10]. The amount of SCFA found in human ileum is approximately tenfold lower than in the colon, ranging from 7 to 20 mM [10].

SCFA play important roles in human health and diseases. They are involved in energy metabolism and signal transduction in intestinal epithelial and immune cells, attributed to their function as metabolic substrates for the colonocytes and ligands for surface receptors of epithelial and immune cells, respectively [11]. SCFA can also inhibit histone deacetylases (HDACs), regulating innate and adaptive immune responses at multiple levels, including Toll-like receptor (TLR) and interferon (IFN) signaling, cytokine production, leukocyte adhesion, and migration [12,13]. In clinical studies SCFA positively influence the treatment of ulcerative colitis (UC), Crohn’s disease (CD), and antibiotic-associated diarrhea [14]. Moreover, it has been shown that SCFA also modulate the replication, colonization, and virulence gene expression of enteric pathogens such as EHEC, *Klebsiella,* and *Salmonella* in vivo and in vitro [15,16,17]. The regional concentration and composition of SCFA in the gastrointestinal tract may serve as environmental cues that differentially regulate virulence gene expression [15,16]. For example, genes involved in EHEC flagella biosynthesis and motility are upregulated by SCFA simulating the small intestine, but down regulated by SCFA simulating the large intestine [16]. Similarly, the expression of virulence genes in *Salmonella* encoding invasion of epithelial cells and survival within macrophages are increased by ileal SCFA, but inhibited by colonic SCFA [16].

Inflammatory bowel disease (IBD) and colorectal cancer (CRC) are associated with changes in the composition and function of intestinal microbiota, i.e., dysbiosis [18,19]. Patients with IBD or CRC are depleted in bacteria that produce SCFA, in particular the members of butyrate-producing bacteria in the phylum Firmicutes, such as *Faecalibacterium prausnitzii*, *Eubacterium rectale,* and *Roseburia intestinalis* [20,21]. On the other hand, increased abundance of *E. coli,* specifically the adherent and invasive *E. coli* (AIEC) pathotype, has been consistently associated with intestinal inflammation across species, particularly ileal Crohn’s disease (ICD) [22,23], Granulomatous colitis (GC) in dogs [24], and murine models of IBD [25]. The reason for higher prevalence of AIEC in inflamed ileal vs colonic mucosa is unclear, but may relate to differences in the chemical and bacterial microenvironment [26,27,28]. It has recently been shown that the ability of CD-associated *E. coli* to utilize propionate and acetate can impact their ability to colonize the intestines of mice [29,30]. As pathosymbionts, AIEC can invade and persist intracellularly within cultured epithelial cells and macrophages [31,32], and have been causally associated with intestinal inflammation in dogs with Granulomatous colitis and IBD susceptible mice [24,25]. Moreover, invasive *E. coli* and *E. coli* that can induce DNA damage have been identified in patients with CRC [33,34] and promote tumorigenesis in murine models [35]. These results imply that *E. coli* may play an important role in the pathogenesis and development of IBD and CRC. Selectively targeting *E. coli* to reduce their growth and virulence could be an important strategy to break the self-perpetuating cycle of dysbiosis and inflammation in patients with IBD, and to prevent the formation and development of CRC in patients with UC. Resistance to conventional antimicrobials targeting *E. coli* is emerging, with rates up to 52% reported in CD-associated *E. coli* [36,37], and up to 50% multidrug resistance (MDR) and fluroquinolone resistant in dogs with *E. coli*-associated granulomatous colitis [38,39], necessitating the development of alternate strategies.

Given the effect of SCFA on enteropathogens [15,16], we sought to investigate their impact on the growth and virulence of pathosymbiont *E. coli* associated with IBD and CRC, and their role in regulating host responses to *E. coli* infection in vitro. We modeled our experimental parameters (SCFA and pH) on the in vivo chemical microenvironment of the human ileum and colon, and used *E. coli* strains isolated from patients with CD and CRC, and animals with inflammatory diseases. Our results reveal that SCFA have a multi-faceted effect, promoting growth and virulence of *E. coli* at ileal concentrations and pH, but suppressing growth, virulence, and pro-inflammatory responses at colonic concentrations and pH. Our findings support a role of the regional chemical microenvironment in regulating the growth and virulence of pathosymbiont *E. coli*, providing an explanation for the differential association of AIEC with ileum and colon, and unique opportunities for therapeutic intervention (prebiotic, probiotic and SCFA) to suppress the growth and virulence of dysbiotic pathosymbiont *E. coli*.

## 2. Results

### 2.1. SCFA Modulate E. coli Growth In Vitro

The effects of SCFA on *E. coli* were evaluated at concentrations of 12, and 60–123 mM, to simulate the ileum and colon, respectively (Table 1). *E. coli* strains were isolated from people with IBD (UC, CD), CRC and CD-spondyloarthritis, dogs with granulomatous colitis, and murine models of intestinal inflammation (Table 2). Our primary focus was on human strains characterized as symbionts (CUT75), or pathosymbionts with a CD-associated AIEC pathotype or CRC-associated *pks* genotoxicity.

#### 2.1.1. Ileal SCFA (i-SCFA) Promote *E. coli* Growth

**Figure 1:**
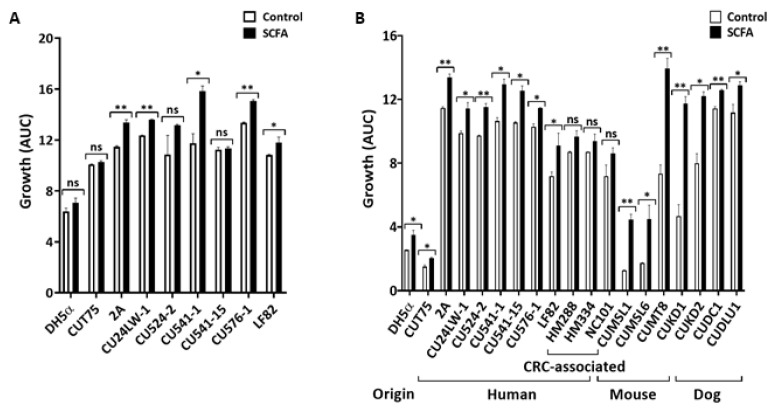
Ileal SCFA promote *E. coli* growth in vitro. Overnight culture of *E. coli* was diluted (1:100) into fresh LB (**A**) or M9 (**B**) media containing either 12 mM NaCl (Control) or SCFA at pH = 7.4. To simulate the enteric luminal environment, bacteria were grown under microaerophilic conditions for 24 h at 37 °C. Data from 3 independent experiments; Mean ± SE. * *p* < 0.05; ** *p* < 0.01; ns = not significant.

SCFA are detected in the distal ileum at total concentrations of 10 to 20 mM [10]. The pH in the distal ileum is 7.4 in health and disease [45,46]. The influence of SCFA on *E. coli* growth under the ileal conditions (12 mM total SCFA, pH = 7.4; see Table 1) was examined in complex medium (Luria-Bertani, or LB) and chemically defined medium M9 (see Materials and Methods). To simulate the enteric luminal environment, bacteria were grown under microaerophilic conditions for 24 h at 37 °C. i-SCFA enhanced (*p* < 0.05) the growth of *E. coli* (Figure 1A,B) in both media: 5/9 strains in LB (Figure 1A) and 16/19 strains in M9 medium (Figure 1B). The degree of stimulation ranged from 10 to 60% and was strain specific. Three of the four mouse strains (CUMSL1, CUMSL6, and CUMT8) grew much better (>50%; *p* < 0.05) in the presence of i-SCFA compared to NaCl controls (Figure 1B). In addition, i-SCFA stimulated-*E. coli* growth was media independent, and unaffected by the origins and disease association of *E. coli.*

#### 2.1.2. Colonic SCFA (c-SCFA) inhibit *E. coli* Growth

**Figure 2:**
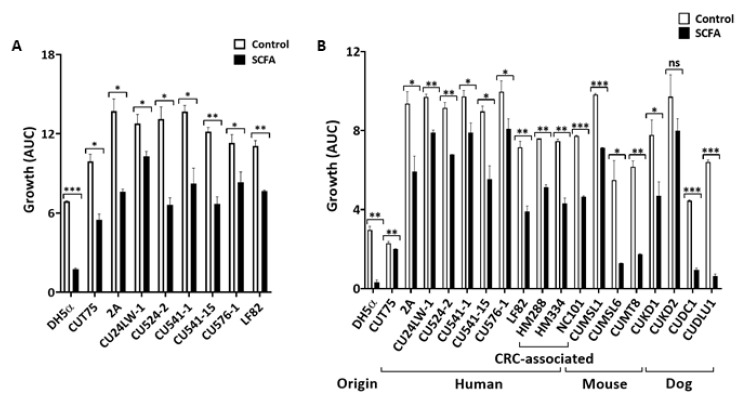
Colonic SCFA inhibit *E. coli* growth in vitro. *E. coli* were cultured in LB (**A**) or M9 (**B**) media containing either 123 mM NaCl (Control) or SCFA at pH = 6.5. Growth conditions were the same as for Figure 1. Data from three independent experiments; Mean ± SE. * *p* < 0.05; ** *p* < 0.01; *** *p* < 0.001; ns = not significant.

Colonic concentrations of SCFA range from 60–150 mM in a healthy gut [9,10]. The physiological pH of the colon is between 5.6 and 6.7 [45,46]. To investigate the effect of SCFA on *E. coli* growth under the colonic conditions, we evaluated total SCFA at a concentration of 123 mM (65 mM acetate, 29 mM propionate, and 29 mM butyrate) and pH = 6.5 to simulate the in vivo milieu [10]. In both complex (LB, Figure 2A) and chemically defined (M9, Figure 2B) media, c-SCFA inhibited the growth of *E. coli* (*p* < 0.05) from human, mice, and dogs (except canine GC CUKD2 in M9 media, *p* = 0.194). The degree of inhibition varied by strain and was independence of media type. Non-pathogenic DH5α, canine GC-AIEC CUDC1 and CUDL1, and murine AIEC CUMSL1 and CUMSL6 were highly sensitive to c-SCFA (Figure 2B), with average growth inhibited >80% (*p* < 0.05) under the same conditions.

Further analysis with different C- and N-sources in M9 background revealed that the inhibition of *E. coli* growth by c-SCFA was independent of C- and N-sources (data not shown).

#### 2.1.3. Inhibition of Growth by c-SCFA is pH-Dependent

**Figure 3:**
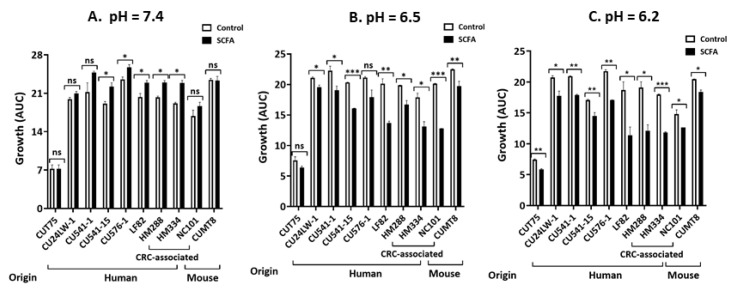
Inhibition of *E. coli* growth by colonic SCFA is pH dependent. LB broth was adjusted to pH = 7.4, 6.5 or 6.2 with HEPES (pH = 7.4), MOPS (pH = 6.5) or PIPES (pH = 6.2). The growth conditions were the same as described in Figure 1. Data from 3 independent experiments; Mean ± SE. * *p* < 0.05; ** *p* < 0.01; *** *p* < 0.001; ns = not significant.

Colonic pH ranges from 5.6 near the cecum to 6.6 in the left colon vs 7.4 in the distal ileum [46]. To determine the influence of regional pH on the inhibitory effect of c-SCFA on *E. coli* growth, we used buffered-LB broth (containing 100 mM HEPES for pH = 7.4, 100 mM MOPS for pH = 6.5, or 100 mM PIPES for pH = 6.2 medium, respectively) ± c-SCFA. At pH = 7.4, none of the 10 *E. coli* strains were inhibited by c-SCFA (Figure 3A), rather 50% grew better in the presence of c-SCFA (*p* < 0.05) (Figure 3A). In contrast, the growth of pathosymbiont *E. coli* strains were inhibited (*p* < 0.05) at pH ≤ 6.5 (Figure 3B,C), with the exception of AIEC CU576-1 (*p* = 0.061) in medium pH = 6.5 (Figure 3B). The growth of symbiont *E. coli* CUT75 was less affected at pH = 6.5 (*p* = 0.119) but was reduced to 79% of control (*p* < 0.01) at pH = 6.2 (Figure 3C).

#### 2.1.4. Inhibition of *E. coli* Growth by Acetate, Propionate, and Butyrate

**Figure 4:**
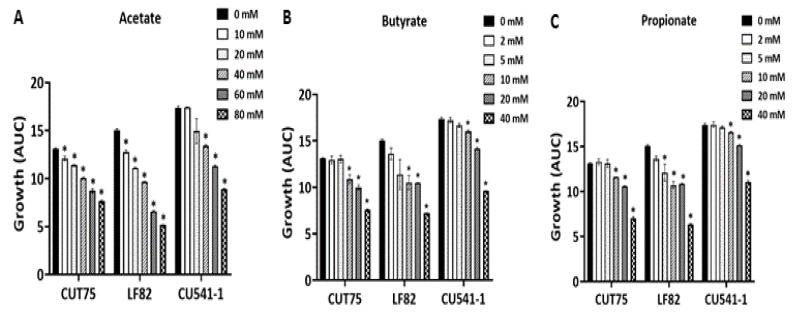
Inhibition of *E. coli* growth by individual SCFA. *E. coli* was grown in LB broth at pH = 6.5, with added individual SCFA as shown in the figures. The osmolarity was balanced out with NaCl. Growth conditions were the same as for Figure 1. Data from three independent experiments; Mean ± SE. * *p* < 0.05.

Depending on diet, the physiological concentration of SCFA in the colon ranges from 40 to 70 mM acetate, 15 to 30 mM propionate, and 15 to 30 mM butyrate [10]. The minimal inhibitory concentration (MIC) of each individual SCFA required to inhibit *E. coli* growth at colonic pH = 6.5 was determined by titration experiments. We observed dose-dependent inhibition of *E. coli* growth by all three SCFAs (Figure 4). Acetate (Figure 4A) inhibited bacterial growth at concentrations ≥10 mM for symbiont CUT75 and AIEC LF82, but ≥40 mM for AIEC CU541-1 (*p* < 0.05). Butyrate (Figure 4B) and propionate (Figure 4C) had the same MIC value of 10 mM for all three *E. coli* strains (CUT75, LF82, and CU541-1), though propionate appeared to inhibit AIEC LF82 growth (*p* < 0.05) at 5 mM (Figure 4C).

### 2.2. c-SCFA Inhibit Virulence Gene Expression in E. coli

Based on their effects on *E. coli* growth (Figure 2, Figure 3 and Figure 4), we speculated that c-SCFA may also impact virulence gene expression in pathosymbiont *E. coli*. We evaluated a panel of 11 virulence genes (Table 3) in CD-AIEC and CRC-pks^−/+^ *E. coli* ± c-SCFA. To achieve an adequate yield of bacteria for total RNA isolation (see Methods) at mid-log phase, we used SCFA at sub-maximal inhibitory concentration (65 mM vs.123 mM) with the same molar ratio (Table 1). Virulence gene expression, determined by qRT-PCR (Table 3; Primers in Table 4), showed that c-SCFA treatment down regulated numerous virulence genes (*p* < 0.05), especially those associated with motility (*fliC*), adhesion and invasion (*fimH, ompC, yfgL,* and *lpfA*), stress (*htrA*) and genotoxicity (*pks*) for majority of *E. coli* strains. For instance, the motility gene *fliC* was down regulated in 11/15 *E. coli* strains (*p* < 0.05), and the adhesin gene *fimH* in 9/15 strains (*p* < 0.05) (Table 3).

### 2.3. SCFA Modulate E. coli Motility

**Figure 5:**
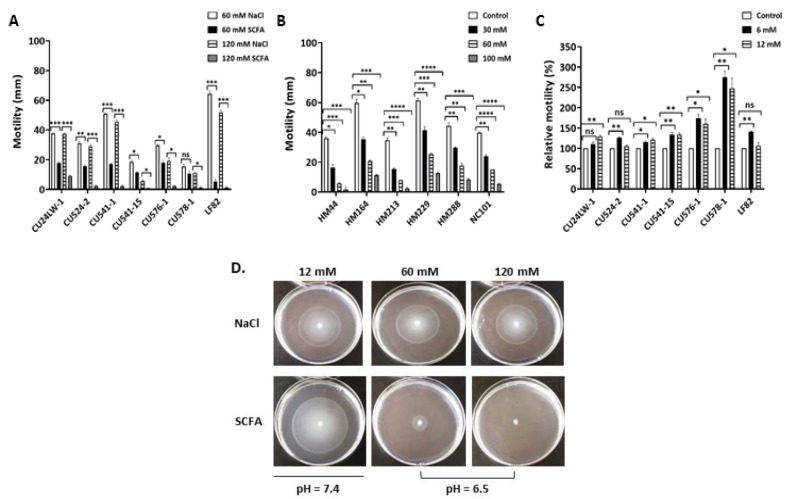
*E. coli* motility was inhibited by c-SCFA but promoted by i-SCFA. Soft agar was used to measure the swarming ability of *E. coli* under either colonic ((**A**), (**B**) and (**D**); pH = 6.5) or ileal ((**C**) and (**D**); pH = 7.4) conditions. D. Images of swarming AIEC CU541-1. Mean ± SD; * *p* < 0.05; ** *p* < 0.01; *** *p* < 0.001; ns = not significant.

Bacterial motility is involved in the virulence of pathogens [47] and directly correlates with the ability of AIEC to invade epithelial cells [28]. Recent studies of CD-*E. coli* have linked motility to the AIEC pathotype [28,48]. The flagellin protein FliC plays an essential role in bacterial motility. The functional consequence of down regulation of *fliC* by c-SCFA (Table 3) was evaluated by motility assays on sloppy agar containing different concentrations of total SCFA (Figure 5A–D). Non-motile, non-AIEC symbiont CUT75 was excluded from the analysis. The motility of CD-associated *E. coli* was reduced significantly (*p* < 0.05) compared to the NaCl controls at pH 6.5, even at 60 mM level of c-SCFA (Figure 5A,D), with the exception of AIEC CU578-1 (*p* = 0.215). At 120 mM, c-SCFA reduced *E. coli* motility to <5% (Figure 5A,D), except for AIEC CU24LW-1 (<25%). Prototypical AIEC LF82 was greatly impacted (Figure 5A), with a >90% reduction of motility at 60 mM c-SCFA. We observed similar dose-dependent inhibition for CRC-*E. coli* (Figure 5B) under the same assay conditions. At 30 mM c-SCFA, more than 30% of inhibition was obtained across all strains (Figure 5B), while the motility of CRC-*E. coli* was completely inhibited at 123 mM c-SCFA (data not shown).

In contrast, i-SCFA stimulated (*p* < 0.05) *E. coli* motility (Figure 5C, and the left column in Figure 5D), particularly the AIEC CU576-1 and CU578-1. At the physiological concentrations (6 to 12 mM), i-SCFA stimulated the motility of AIEC CU576-1 up to 1.5 fold, and CU578-1 up to 2.5 fold (*p* < 0.05).

### 2.4. c-SCFA Inhibit Type 1 Pili FimH-Mediated Yeast Agglutination

**Figure 6:**
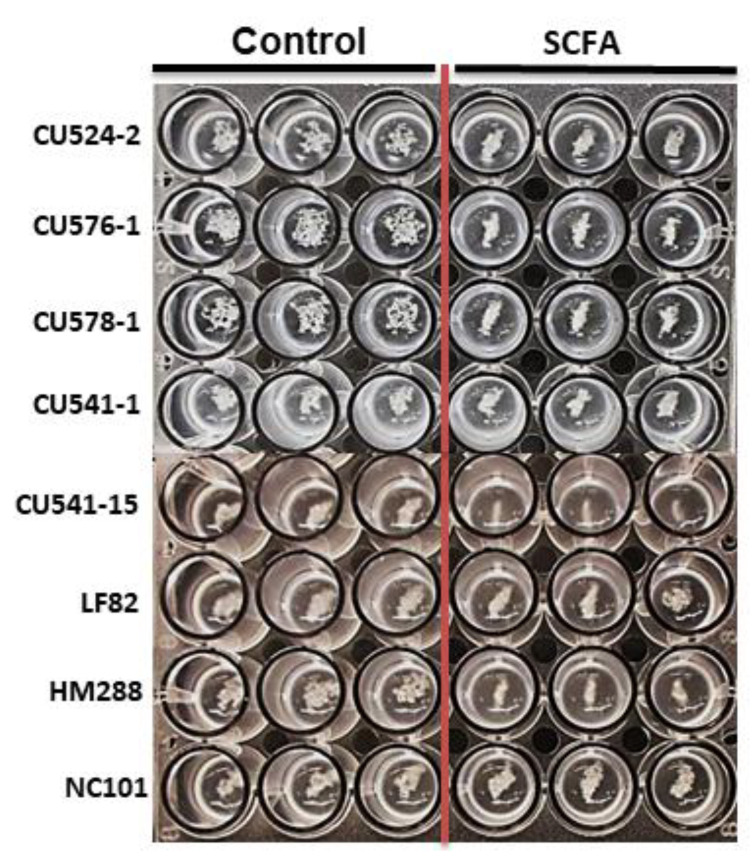
c-SCFA inhibit yeast agglutination by *E. coli*. *E. Coli* were grown in LB media (pH = 6.5) with either 123 mM NaCl (control) or SCFA at 37 °C for 18 h. After centrifugation, the cell pellets were resuspended in PBS, followed by mix with equal volume of yeast suspension at the same optical density (OD^600^ = 5.2) (see Methods).

*E. coli* type I fimbrial protein, FimH, can bind to the mannose residues on yeast cell surface, and subsequently initiate yeast cell agglutination [49]. This activity of FimH also mediates the adherence and colonization of *E. coli* to the intestinal epithelial cells [50,51,52] and stimulates TLR4 [53]. We visualized the functional consequences of c-SCFA on *fimH* gene expression with yeast agglutination assays. After pretreatment with 123 mM c-SCFA or NaCl (control) in LB broth at pH 6.5, *E. coli* were mixed with equal amount of yeast cells. Pretreatment of *E. coli* with c-SCFA reduced agglutination (Table 5 and Figure 6) compared to NaCl controls. c-SCFA induced a 2-fold or greater reduction in yeast cell agglutination for the majority (5/8) of *E. coli* strains (Table 4). The biggest reduction was seen with CD-AIEC CU541-15 and CRC-*pks*^−^ HM288 (Table 5; Figure 6).

### 2.5. c-SCFA Inhibit E. coli Adhesion and Invasion of Intestinal Epithelial Cells

**Figure 7:**
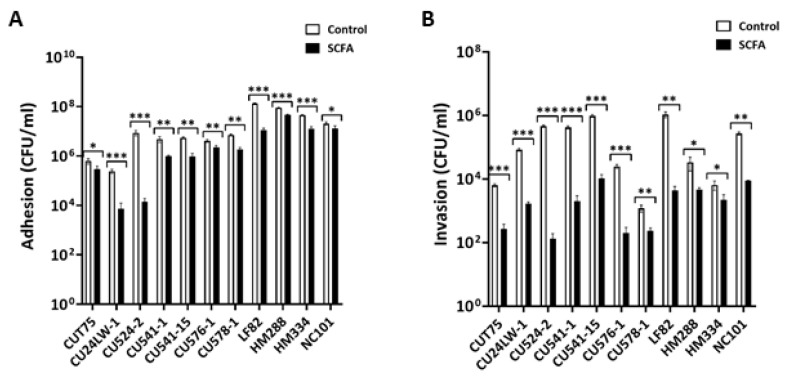
c-SCFA inhibit *E. coli* adhesion and invasion of intestinal epithelial cells. Overnight culture of *E. coli* was added to the monolayer of intestinal epithelial cells (Caco-2) in the presence of 65 mM NaCl (Control) or SCFA at pH = 6.5. The number of adherent or invasive *E. coli* were determined by quantitative plating method (see Methods). (**A**). Adhesion to Caco-2 cells; (**B**). Invasion of Caco-2 cells. Data from three independent experiments; Mean ± SD. *p < 0.05; **p < 0.01; ***p < 0.001.

c-SCFA down regulated a number of virulence genes (*fimH, ompC, nlpL,* and *lpfA)* involved in the process of adhesion and invasion [54,55,56,57] (Table 3), suggesting that c-SCFA would reduce the infection of intestinal epithelial cells by *E. coli*. In the presence of 65 mM c-SCFA, the ability of *E. coli* to adhere to and invade Caco-2 epithelial cells was significantly reduced (*p* < 0.05; Figure 7A,B). The impact of c-SCFA was greater (*p* = 0.0073) on invasion than adhesion compared with controls (Figure 7A vs. Figure 7B), particularly for CD-AIEC CU524-2, CU541-1, CU541-15, CU576-1, CU578-1, and LF82. Under identical conditions, the average inhibition of c-SCFA was 71% for adhesion and 93% for invasion (Figure 7A vs Figure 7B).

### 2.6. c-SCFA Inhibit Host Proinflammatory Responses

#### 2.6.1. c-SCFA Inhibit NF-kB Signal Transduction

**Figure 8:**
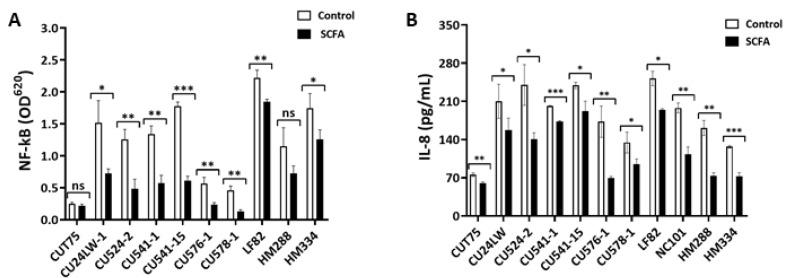
c-SCFA inhibit NF-*κ*B pathway activation and IL-8 production in the host cells. (**A**). Hek-Blue cells were infected with *E. coli* in the presence of 123 mM NaCl or SCFA at pH 6.5 (see Methods). At 24 h post infection, the supernatant of the infected cells was used to detect the reporter protein (secreted alkaline phosphatase, SEAP) production. (**B**). Caco-2 cells were infected by *E. coli* for 3 h in the presence of either NaCl or c-SCFA (65 mM) at pH = 6.5. The supernatant of infected Caco-2 cells was used for IL-8 detection using ELISA method. Data from three independent experiments; Mean ± SE. *p < 0.05; **p < 0.01; ***p < 0.001; ns = not significant.

The nuclear factor, NF-*κ*B, is a family of regulators controlling multiple cellular inflammatory responses through signal transduction. It is present in all types of cells, and can be induced by pathogen infection [58]. To determine the effect of c-SCFA on host epithelial responses to *E. coli* infection, we used HEK-Blue KD-TLR5 cells to detect the NF-*κ*B pathway activation [59]. In the presence of 123 mM c-SCFA, the activation of NF-*κ*B pathway by most *E. coli* strains was inhibited (*p* < 0.05), except for non-pathogenic CD-*E. coli* CUT75 and the *pks*-negative CRC-*E. coli* HM288 (Figure 8A). The inhibition ranged from 17 to 70% compared to controls for all, except non-pathogenic *E. coli* CUT75, which was minimally able to activate NF-*κ*B.

#### 2.6.2. c-SCFA Inhibit IL-8 Secretion by Epithelial Cells

SCFA interact with intestinal epithelial cells and modulate immune responses in the gut [13]. IL-8 is a pivotal chemokine produced by the gut epithelial cells during pathogen infection, and its production is down stream of the NF-*κ*B signal transduction pathway [59]. We measured the levels of IL-8 produced by Caco-2 cells after infection by *E. coli* in the presence or absence of c-SCFA under colonic conditions. c-SCFA inhibited IL-8 secretion (*p* < 0.05) induced by all *E. coli* strains tested (Figure 8B), including CD-AIEC and CRC-associated *E. coli*. The inhibition ranged from 20 to 60% depending on strains (Figure 8B).

## 3. Discussion

Intestinal SCFA, predominantly acetate, propionate, and butyrate, are by-products of bacterial fermentation [1]. Concentrations of SCFA are 10-fold higher in the colon than the ileum, with concordant differences in luminal pH of 7.4 in the ileum and 5.6–6.7 in the colon [9,10,45]. We sought to determine the effects of SCFA in the context of region-specific variations in the chemical microenvironment (SCFA and pH) on the growth and virulence of pathosymbiont *E. coli* isolated from people with Crohn’s disease (AIEC pathotype) and CRC (*pks* genotoxicity), dogs with granulomatous colitis (AIEC pathotype), and mice with intestinal inflammation (AIEC pathotype). We also examined the impact of SCFA on host-*E. coli* inflammatory responses.

We found that SCFA affect the growth of pathosymbiont *E. coli* in a concentration and pH dependent fashion, with colonic [SCFA] at colonic pH suppressing growth, and ileal [SCFA] at ileal pH favoring growth. The effect was largely independent of *E. coli* pathotype, disease association, species of origin, and type of media, supporting a direct effect of SCFA and pH. The concentrations and proportions of SCFA and pH levels we used to model the ileal and colonic microenvironment were selected to be physiologically relevant for people: i-SCFA (12 mM with a molar ratio of 8:2.5:1.5 for acetate, propionate, and butyrate, respectively), c-SCFA (60 to 123 mM with a ratio of 65:29:29 for acetate, propionate, and butyrate, respectively), and pH = 6.2–7.4 [9,10,40]. MIC values for acetate, propionate, and butyrate were 20 to 40, 10, 10 mM, respectively. To further simulate the enteric microenvironment, *E. coli* were cultured in microaerophilic conditions at 37 °C.

A mechanistic understanding of the interactions between the chemical microenvironment (e.g., SCFA, pH, bile acids), the resident microbiota, and host immune responses in the intact GI tract in health and disease remains to be elucidated. Lower fecal concentrations of butyrate and propionate in patients with IBD vs. controls [60], and acetate in CD vs. UC [60], may reflect decreased microbial production, increased utilization, or a combination of these processes. Region-specific differences in the chemical and microbial microenvironment in the ileum and colon may underlie the phenotypic variation of IBD. Crohn’s ileitis is consistently linked to dysbiosis characterized by an overabundance of *E. coli* and depletion of Firmicutes *(*e.g., *Faecalibacterium prausnitzii)* [18,61], and *E. coli* with an AIEC pathotype have been more frequently isolated from ileal (36.4%) than colonic (3.6%) mucosa of CD patients in some studies [18,62]. Our finding that i-SCFA (12 mM, pH = 7.4) promote the growth of AIEC whereas c-SCFA (123 mM, pH = 6.5) suppress it, suggest region specific differences in the chemical environment may influence colonization by pathosymbiont *E. coli*.

SCFA propionate and acetate have recently been implicated in regulating the growth, colonization, and virulence of AIEC [28,29,30,63]. Propionate-adapted AIEC LF82 more proficiently colonized the colon and ileum, but not the cecum, of mice fed propionate (at levels selected to simulating human gut: 20 mM) than non-propionate adapted LF82 [30]. Acetate utilization has also been linked to enhanced colonization by AIEC NRG857 (LF82-like, B2 O83) in mice, and *E. coli* from CD patients were better able to grow on acetate (K-acetate 0.4% *w/v* in M9 media), but not complex media, than *E. coli* from healthy controls [28].

In contrast to the growth enhancing effects of propionate and acetate, we found that propionate at ≥5 mM and acetate ≥10mM suppressed the growth of AIEC LF82 at colonic pH = 6.5. These differences may reflect the pH dependency of the effects of SCFA we observed, with stimulation of growth with c-SCFA at pH = 7.4 and repression at pH = 6.5. However, since the previous studies were conducted in mice, which have a mean intestinal pH of mice < pH = 5.2, and regional pH = 4.8–5.2 in the ileum, 4.4–4.6 cecum and 4.4–5.02 colon [64], substantially lower than that of humans and the conditions we simulated, it is difficult to reconcile these different outcomes. Differences in methodology, such as growth in the microaerophilic conditions and composition of media, may play a role.

In addition to the effects of SCFA on bacterial growth, their regional concentration and composition throughout the gastrointestinal tract may serve as environmental cues that differentially regulate motility and virulence gene expression [15,65]. We found that i-SCFA (12 mM, pH = 7.4) promote the motility of pathosymbiont *E. coli,* whereas c-SCFA (120 mM, pH = 6.5) suppress motility, virulence gene expression, adhesion and invasion of cultured cells, and pro-inflammatory responses. Our findings parallel those with EHEC and *Salmonella.* For example, genes involved in EHEC flagella biosynthesis and motility are upregulated by SCFA simulating the small intestine, but down regulated by SCFA simulating the large intestine [15]. Similarly, the expression of virulence genes in *Salmonella* encoding invasion of epithelial cells and survival within macrophages are increased by ileal SCFA, but inhibited by colonic SCFA [65].

Transcriptional analysis of *E. coli* grown in c-SCFA revealed consistent down-regulation of virulence genes involved in motility (*fliC*), adhesion and invasion (*fimH, ompC, lpfA*, and *nlpL*), iron acquisition (*chuA*), stress protein (*dsbA* and *htrA*), and colibactin protein (*pks*). Reductions in transcription correlated with reduced functions, e.g., reduced *fliC* gene transcription with decreased motility; reductions in *fimH, yfgL, ompC* and *nlpL* with decreased yeast agglutination, adhesion and invasion of intestinal epithelial cells. c-SCFA also reduced the ability of CD- and CRC-*E. coli* induced activation of NF-*ĸ*B, and the secretion of IL-8 by Caco-2 epithelial cells. NF-*κ*B regulates a large array of genes associated with immune and inflammatory responses [58] and controls in part the secretion of IL-8, which is upregulated in patients with IBD and CRC [66,67]. Our findings support a direct role of the chemical microenvironment (SCFA, pH) in modulating crosstalk between pathosymbiont *E. coli* and the epithelium and pro-inflammatory signaling.

The efficacy of SCFA against enteropathogens is exemplified by the use of propionate to suppress *Salmonella* associated disease in poultry [68,69]. In the context of the colonic environment (65–123 mM SCFA, pH = 6.5), we found that SCFA mixtures containing 29 mM propionate markedly suppressed parameters associated with virulence of pathosymbiont *E. coli* associated with intestinal inflammation across species. However, in the context of the ileal environment, we found that i-SCFA (containing 2.5 mM propionate, 8 mM acetate, 1.5 mM butyrate) stimulated growth and motility of pathosymbiont *E. coli*, including LF82. Previous studies have shown that propionate can enhance the ability of AIEC to adhere to (2/5 AIEC strains) and invade (3/5 AIEC) Caco-2 cells [30], and increase transcription of the *eut* operon and ability to utilize ethanolamine [29], which is linked to virulence in a number of enteropathogens [70,71]. Motility has been reported to correlate with the degree of invasion in vitro by AIEC, murine colonization by AIEC NRG857, and the isolation of *E. coli* from CD patients vs healthy controls [28]. These findings point to complex multifactorial interactions of the metabolism, growth, and virulence of pathosymbiont *E. coli*, region specific luminal microenvironment and host.

Our investigations of the effects of SCFA on virulence extended to the genotoxic effects of *E. coli* associated with CRC. The polyketide synthase gene (*pks*) is responsible for the formation of colibactin, which is mutagenic [35,72]. We found that c-SCFA down-regulate *pks* transcription in *E. coli* NC101, which induces inflammation-associated CRC in mice [35], and *E. coli* isolated from patients with CRC [43]. These findings suggest that the chemical environment of the healthy colon may restrict the ability of *pks*+ CRC-*E. coli* to grow and produce colibactin. Recent studies in patients with CRC reveal a correlation between the loss of *Bifidobacterium* and reduced levels of total SCFA, especially butyrate, in CRC patients vs healthy controls [73]. While it remains to be established if reduced SCFA leads to proliferation and colibactin production by *E. coli*, it suggests the potential for therapeutic intervention with SCFA, which are known to be protective against the development of CRC [2,12].

The mechanism by which SCFA inhibit bacterial growth is postulated as intracellular acidification caused by uptake of these free acids at acidic pH [17,74]. At acidic pH, undissociated SCFA can freely diffuse through cell membrane and concentrate in bacterial cytoplasm, resulting in reduction of the intracellular pH [17]. Acidified bacterial cells have reduced transmembrane potentials and disrupted cellular biological activities (such as DNA replication), thereby exhibiting low growth phenotype. This explains the results that c-SCFA inhibit *E. coli* growth only at pH ≤ 6.5, but not at pH = 7.4. Sorbara et al. reported that *E. coli* and *Klebsiella* failed to replicate at internal pH = 7 or 7.25, respectively, and an internal pH = 6.75 or 6.5 is bactericidal [17], indicating the importance of luminal pH of the host in bacterial fitness. In the same report [17], the authors also found that at concentrations ≥10 mM, acetate, butyrate, and propionate (>10 mM) were able to reduce the intracellular pH of *K*. *pneumonia* and *E. coli* to pH < 6.7 at low medium pH (pH = 5.75), which resulted in slow growth of these bacteria. These results raise the speculation that when the levels of c-SCFA are reduced due to inflammation-associated loss of commensal bacteria, the luminal pH would rise, and ultimately the luminal microenvironment would change concurrently. These changes could be in favor of pathosymbiont (like AIEC) growth and virulence gene expression, and consequently potentiate inflammation in the intestine.

## 4. Materials and Methods

### 4.1. Bacterial Strains

In this study, we used 22 *E. coli* strains from different origins, including 1 laboratory strain (DH5α), 13 human, 4 mouse, and 4 dog strains (Table 2). The human strains were from the intestinal mucosa of patients with IBD (9 strains) [32,41,42] and CRC (4 strains) [72]. All the IBD strains, except CUT75, have an AIEC pathotype. CUT75 is a non-pathogenic strain from a CD patient [32] and was used as a non-AIEC control in this study. Prototypical AIEC LF82 (kindly provided by Arlette Darfeuille-Michaud) [40] was used as a positive control for AIEC. The 4 CRC-associated *E. coli* strains (Table 2) were kindly provided by Dr. Jonathan Rhodes [72]. The mouse strain NC101 was isolated from the feces of a healthy mouse, and it induces CRC in IL10-/- AOM treated and monocolonized mice [33,74]. CUMSL1 and CUMSL6 were isolated in our laboratory from Agr2^−/−^ mouse ileum provided by Dr. Steven Lipkin. CUMT8 was isolated from mouse ileitis tissue in our laboratory [25]. The 4 dog strains CUDC1, CUDLU1, CUKD1 and CUKD2 were isolated in our laboratory from dog colons with granulomatous colitis (GC) [24,38]. All *E. coli* strains were stored at −70 °C.

### 4.2. Bacterial Culture

A single colony from a fresh Luria-Bertani (LB) agar plate was used to prepare a stock culture for each experiment. Two types of liquid culture media were used in this study, LB and M9 minimal media. M9 minimal medium was made of 1 × M9 salts (33.7 mM Na_2_HPO_4_, 22 mM KH_2_PO_4_, 8.55 mM NaCl, and 19 mM NH_4_Cl), plus 0.1 mM CaCl_2_, 2 mM MgSO_4_, 10 mM KH_2_PO_4_, 50 µM FeSO4, 5 nM vitamin B1, 150 nM vitamin B12, 15 nM adenosylcobalamin, and 15 nM cobinamide dicyanide. Glycerol (20 mM) was used as the carbon source. All *E. coli* strains were first grown in LB broth overnight, then diluted 1:100 in media (LB or M9) supplemented with SCFA or NaCl at concentrations specified under each condition. For all controls, equal amount of NaCl was added in place of the SCFA used in the experiments described.

### 4.3. Chemicals and Stock Solutions

Sodium acetate, sodium propionate, sodium butyrate, and M9 salts were purchased from Sigma-Aldrich (St. Louis, MO). For consistency and reproducibility, total SCFA solutions were premixed as 1 M stock solutions with a molar ratio of 65:29:29 for acetate, propionate, and butyrate, respectively for c-SCFA, or 8:2.5:1.5 for acetate, propionate, and butyrate, respectively for i-SCFA.

### 4.4. Standardized Growth Analysis

*E. coli* were grown in LB broth overnight at 37 °C with shaking. Overnight cultures were diluted 1:100 into fresh LB broth or M9 medium containing NaCl (control) or SCFA at specified concentration in a 100 well-plate (Growth Curve, USA). On top of the growth medium in each well, 75 µL of mineral oil was gently added to achieve a microaerophilic growth environment. Growth of *E. coli* was monitored 24 to 48 h at 37 °C in a BioScreen C system (Growth Curve, USA). The OD^600^ was taken every 15 min by the machine. Growth curves were generated with OD^600^ as the function of time. For easier comparison between SCFA-treated and untreated control samples, the area under each growth curve (AUC) was calculated with Graphpad Prism7.03.

### 4.5. Transcriptional Analysis of Virulence Genes

*E. coli* was grown in media with either c-SCFA (65 mM) or NaCl (65 mM, Control) to mid log phase. Total RNA was extracted using the Qiagen RNAProtect-RNeasy Kit per manufacturer’s protocol. Total RNA was treated with TURBO DNA-Free Kit (Ambion), followed by a two-step qRT-PCR analysis, using Qiagen’s QuantiTect Reverse Transcription Kit and QuantiNova SYBR Green PCR Kit. Eleven genes associated with virulence of IBD- and CRC-associated *E. coli* (see Table 3) were selected for transcriptional analysis with or without SCFA treatment. Primers for these virulence genes are listed in Table 4. *E. coli mdH* was used as the reference gene. Each qPCR reaction contained 1 μL of cDNA, 0.7 μL of each forward and reverse primers (10 μM), 5 µL of 2× SYBR Green Master Mix, 1 μL of QN ROX Reference Dye and 2.3 μL of nuclease-free water to make the total volume of 10 µL. The reaction was run with ABI7000 (Applied Biosystems). The comparative quantification (ΔC_t_) method was used to determine the up- or down-regulated genes. The relative change of a targeted gene expression was calculated by using the equation RQ = 2^−ΔΔCT^.

### 4.6. Motility Assay

*E. coli* was grown overnight at 37 °C in LB broth. Soft agar plates (1% tryptone, 0.5% NaCl, 0.25% agar) were prepared the day before assay. Sterile 1 M SCFA or NaCl stock solution was added into the agar right before pouring the plates. The control plates contain the same amount of NaCl as the total SCFA. There were three replicates per treatment. The overnight cultures of *E. coli* were transferred (3 µL) on to the center of each plate, followed by incubation of the plates at 37 °C for 10 h. *E. coli* motility was quantified by measuring the diameter of the circular swarming area formed by the growing motile bacteria. *E. coli* T75 and HM334 were found to be non-motile and excluded from this assay.

### 4.7. Mammalian Cell Culture Conditions

Human colonic epithelial cell line Caco-2 (ATCC HTB-37) cells were obtained from the American Type Culture Collection. Caco-2 cells were maintained at 37 °C with 5% CO_2_ in Dulbecco’s modified Eagle’s medium (DMEM) supplemented with 15% fetal bovine serum (Gibco, Rockville, MD, USA).

### 4.8. Yeast Agglutination Assay

*E. coli* was cultured in LB ± 123 mM c-SCFA for 18 h at 37 °C. After centrifugation at 3000× *g* for 15 min at 4 °C, the pellet was resuspended in PBS at OD^600^ = 5.2. Yeast cells were suspended in PBS at OD^600^ = 5.2 on the day of assay. The *E. coli* suspension (100 µL) was mixed with equal volume of the yeast cell suspension in a well of 48-well plate. The plate was kept on ice and rocked for 30 to 60 min at 20 rpm.

### 4.9. E. coli Adhesion and Invasion of Cultured Epithelial Cells

*E. coli* was cultured overnight in LB at 37 °C with shaking. Bacterial pellets were re-suspended in PBS before dilution in cell culture media ±65 mM c-SCFA to an m.o.i (multiplicity of infection) of 10. Caco-2 cells were infected with bacteria using the same procedures as described by Zhang et al. [59]. At 3 h post infection, cells were washed 3× with PBS, and lysed with 1% Triton X-100. Serial dilutions of the lysates were made in PBS and plated on LB agar. The total number of colonies recovered was used to calculate the number of adherent bacteria. For invasion assays, cells were treated with gentamicin (100 µg mL^−1^) for one hour after initial infection and 3× wash with PBS to kill extracellular bacteria. Cells were then washed 3× after gentamicin treatment, lysed, and plated as described above.

### 4.10. NF-ĸB Activation Assay

HEK-Blue KD-TLR5 cells were used to detect the induction of NF-ĸB by *E. coli* infection, as previously described by Zhang et al. [59]. Briefly, cells were seeded in 96-well plates at a density of 5 × 10^4^ cells per well. *E. coli* was diluted into fresh cell medium containing either 123 mM NaCl (control) or SCFA at an m.o.i of 200 as 10× inocula, followed by addition of this inoculum (10 µL) into each well containing 100 μL of medium for a final m.o.i of 20. At 3 h post infection, the cell medium was carefully removed from each well, and replaced with 100 µL of fresh medium containing gentamycin (200 μg mL^−1^). At 24 h post infection, the spent medium was collected, and centrifuged at 12,000 rpm for 5 min to remove any particulate matter. QUANTI-Blue Kit (InvivoGen, San Diego, CA, USA) was used to detect the reporter protein SEAP (secreted alkaline phosphatase) following the manufacturer’s instructions. The SEAP activity was detected as optical density at 620 nm.

### 4.11. Proinflammatory Cytokine IL-8 Secretion

Supernatants of Caco-2 (at 3 h post infection) cell cultures were collected and centrifuged to remove any cells or cell debris. The concentrations of IL-8 secreted by Caco-2 cells were analyzed by ELISA methods, using the Human IL-8 Antibody Pair Kit (Invitrogen) as per the manufacturer’s instructions.

### 4.12. Statistical Analysis

Differences in growth, gene expression, motility, adhesion, invasion, and cytokine production between control and SCFA-treated samples were analyzed by 2-way ANOVA with Dunnett’s test for multiple comparisons. All statistical analyses were performed with GraphPad Prism 7.03 software and *p* < 0.05 was considered significant.

## 5. Conclusions

In conclusion, our data reveal a multifaceted and previously unrecognized role of the regional chemical microenvironment (SCFA and pH) on growth and virulence of IBD- and CRC-associated *E. coli,* and on pro-inflammatory pathosymbiont-host interactions. Our findings provide novel insights and opportunities for therapeutic intervention in people and companion animals centered on restraining the growth and virulence of pathosymbiont *E. coli* through modification of the luminal SCFA and pH.

## Figures and Tables

**Table 1:** Experimental conditions.

Condition	Total SCFA	Molar Ratio (Acetate:Propionate:Butyrate)	Reference
Ileal, pH = 7.4	12 mM	8:2.5:1.5	[10]
Colonic, pH = 6.5	60 to 123 mM	65:29:29	[10]

**Table 2:** *E. coli* strains used in the study.

Strain	Source	Phylogroup	AIEC	PKS	Reference
DH5α	ATCC	A	-	-	-
CUT75	CD mucosa	A	-	-	[32]
LF82	CD mucosa	B2	+	-	[40]
CU24LW-1	CD mucosa	A	+	-	[41]
CU524-2	CD mucosa	B1	+	-	[32]
CU541-1	CD mucosa	B1	+	-	[32]
CU541-15	CD mucosa	B1	+	-	[32]
CU578-1	CD mucosa	D	+	-	[32]
CU576-1	CD mucosa	D	+	-	[32]
2A	CD-SpA mucosa	B2	+	-	[42]
HM44	CRC mucosa	B2	- ^b^	+	[43]
HM164	CRC mucosa	B2	- ^b^	+	[43]
HM288	CRC mucosa	B2	-	-	[43]
HM334	CRC mucosa	B2	-	+	[43]
NC101 ^a^	Healthy mouse feces	B2	+	+	[35,44]
CUMT8	mouse ileitis tissue	B1	+	-	[25]
CUMSL1	Agr2^−/−^mouse ileum	B2	+	-	this study
CUMSL6	Agr2^−/−^mouse ileum	B2	+	-	this study
CUDC1	GC dog colon	B1	+	-	[38]
CUDLU1	GC dog colon	B1	+	-	[38]
CUKD1	GC dog colon	B2	+	-	[24]
CUKD2	GC dog colon	D	+	-	[24]

^a^ NC101 induces IBD and cancer in IL10-/- mice. ^b^ Because of their cytotoxicity, these strains were not tested for AIEC characteristics.

**Table 3:** SCFA regulates virulence gene transcription in *E. coli.*

Gene Function	Gene Name	Fold Change (2^−ΔΔ^^Ct^)												
		Healthy Control	CD-associated *E. coli* (AIEC)							CRC-associated *E. coli*				
		CUT75 ^b^	LF82	CU24LW-1	CU524-2	CU541-1	CU541-15	CU576-1	CU578-1	NC101 ^a^	HM44	HM164	HM334	HM288
Motility	*fliC*	0.34	0.31 *	0. 73 *	0.51 *	0.41 *	0.76	0.40 *	0.17 *	0.07 *	5.73 *	0.058 *	0.50 *	0.91
Adhesion and invasion	*fimH*	0.65 *	0.63 *	2.38 *	0.37 *	0.28 *	0.79 *	0.46	0.44 *	0.63	1.25	0.43 *	0.67 *	1.04
	*ompC*	1.48 *	0.42 *	1.99 *	0.30 *	0.31 *	0.50 *	0.60	0.49 *	0.63	0.22 *	0.74 *	0.96	0.33 *
	*yfgL*	1.1	0.77 *	0.44 *	0.62 *	0.34 *	0.70	0.37 *	0.44 *	0.32 *	0.23 *	0.45 *	0.52 *	0.60 *
	*nlpL*	0.73	0.35 *	2.46 *	1.60 *	0.52 *	1.37	1.19	0.26 *	0.54 *	0.51 *	2.05 *	1.12	1.62 *
	*lpfA141*	na	0.39 *	na	na	na	na	na	na	na	na	na	na	na
	*lpfA154*	na		5.54 *	0.90	0.24 *	0.85 *	0.54	0.48 *	na	na	na	-	-
Stress	*htrA*	0.49 *	0.91 *	0.40 *	0.58 *	0.35 *	0.45 *	0.40 *	0.32 *	0.59 *	0.24 *	0.35 *	0.36 *	0.48 *
	*dsbA*	1.37 *	1.05	1.75 *	1.21	0.40 *	1.01	0.44 *	0.47 *	0.40 *	0.23 *	0.71 *	0.51 *	0.71 *
Iron acquisition	*fyuA*	na	1.02	na	na	na	1.10	1.00	1.08	0.92	0.43 *	1.200	0.44 *	1.47
	*chuA*	na	0.33	na	na	na	na	2.29	1.17	0.73	0.57	0.62 *	0.55 *	1.58 *
Genotoxicity	*pks*	na	na	na	na	na	na	na	na	0.54	0.058 *	0.69	0.64 *	na

* *p* < 0.05; na: gene absent in the strain.; -: not tested; ^a^: NC101 induces IBD and cancer in IL10-/- mice. Therefore, it belongs to both groups; ^b^: CUT75 is a non-mobile symbiont *E. coli.*

**Table 4:** Primer sequences.

Gene	Protein	Function	Primer Sequences (5′→ 3′)
fliC *	Flagellin	Motility	F1: CAGCCTCTCGCTGATCACTC
			R1: CCCGCTGCGTCATCCTTCGC
			F2: CTGTCGCTGTTGACCCAGAA
			R2: TGACCTGCTGCGTCATCTTT
*fimH*	Type 1 fimbrial subunit	Adhesion	F: CTTATGGCGGCGTGTTATCT
			R: CGGCTTATCCGTTCTCGAATTA
*ompC*	Outer membrane protein C	Outer membrane protein	F: GGTGGTCTGAAATACGACGCTAAC
			R: GTCGAACTGGTACTGAGCAACAGC
*yfgL*	Lipoprotein	Invasion	F: CCGGTGGTCAGCGACGGTCTGG
			R: CGCCACGCAAAGAGAGCGAAGGC
*nlpL*	Lipoprotein	Invasion	F: GGCTCAAGGCGGACGCAACGG
			R: GAACAGTGCCGTGGCGCTGTCC
*lpfA_141_*	Long polar fimbrial protein A	M cell translocation	F: GCTGATGCAGGCGACGGTTCTG
			R: CACAGACTTGTTCACCTGGCCC
*lpfA_154_*	Long polar fimbrial protein A	M cell translocation	F: CAGGTGTAGGTAGTCTGGCGTC
			R: GGTCGCCGTCGCCGCCAGGCGC
*htrA*	Periplasmic protease	Stress protein (Macrophage survival)	F: CGCAGATGGTGGAATACGGCCAGG
			R: CCTGGCTTACGAAAGCACCGCGC
*dsbA*	Disulfide oxidoreductase	Oxidoreductase (Macrophage survival)	F: GGCGCAGTATGAAGATGGTAAAC
			R: TTCAAACTGATAGCAGTGCGG
*chuA*	Outer membrane heme/hemoglobin receptor	Heme iron acquisition	F: CGGCGACAACTATGTCGTATAA
			R: TAGGCCACATCAAGGCTAAAC
*fyuA*	Ferric Yersiniabactin uptake receptor	Iron acquisition	F: TCGTCGCCGAGAAATCCATCAACT
			R: AAAGCTGCATGTCTTTGGTGTGGG
*pks*	Polyketide synthetase	Genotoxin production	F: ATCTTTCCGCCTAACCCGA
*mdH*	Malate dehydrogenase	Reference	F: CAACTGCCTTCAGGTTCA R: GCGTTCTGGATGCGTTTGGT

* *fliC* primers: F1 and R1 were designed for all *E. coli* strains, except for LF82, CU524-2, and CU24LW-1; F2 and R2 were for *E. coli* LF82, CU524-2, and CU24LW-1.

**Table 5:** Yeast agglutination scores *.

Name	Control	SCFA Treated
CU524-2	4	2
CU576-1	4	2
CU578-1	4	2
CU541-1	3	2
CU541-15	3	1
LF82	3	2
HM288	4	1
NC101	3	2

* The yeast agglutination scores were made based on the degree of aggregation in Figure 6
